# Current automated 3D cell detection methods are not a suitable replacement for manual stereologic cell counting

**DOI:** 10.3389/fnana.2014.00027

**Published:** 2014-05-07

**Authors:** Christoph Schmitz, Brian S. Eastwood, Susan J. Tappan, Jack R. Glaser, Daniel A. Peterson, Patrick R. Hof

**Affiliations:** ^1^Department of Neuroanatomy, Ludwig-Maximilians-University of MunichMunich, Germany; ^2^MBF BioscienceWilliston, VT, USA; ^3^MBF LaboratoriesWilliston, VT, USA; ^4^Center for Stem Cell and Regenerative Medicine, Rosalind Franklin University of Medicine and ScienceNorth Chicago, IL, USA; ^5^Fishberg Department of Neuroscience and Friedman Brain Institute, Icahn School of Medicine at Mount SinaiNew York, NY, USA

**Keywords:** automated cell segmentation, disector, FARSIGHT, Fractionator, ImageJ, stereology, stem cells

## Abstract

Stereologic cell counting has had a major impact on the field of neuroscience. A major bottleneck in stereologic cell counting is that the user must manually decide whether or not each cell is counted according to three-dimensional (3D) stereologic counting rules by visual inspection within hundreds of microscopic fields-of-view per investigated brain or brain region. Reliance on visual inspection forces stereologic cell counting to be very labor-intensive and time-consuming, and is the main reason why biased, non-stereologic two-dimensional (2D) “cell counting” approaches have remained in widespread use. We present an evaluation of the performance of modern automated cell detection and segmentation algorithms as a potential alternative to the manual approach in stereologic cell counting. The image data used in this study were 3D microscopic images of thick brain tissue sections prepared with a variety of commonly used nuclear and cytoplasmic stains. The evaluation compared the numbers and locations of cells identified unambiguously and counted exhaustively by an expert observer with those found by three automated 3D cell detection algorithms: nuclei segmentation from the FARSIGHT toolkit, nuclei segmentation by 3D multiple level set methods, and the 3D object counter plug-in for ImageJ. Of these methods, FARSIGHT performed best, with true-positive detection rates between 38 and 99% and false-positive rates from 3.6 to 82%. The results demonstrate that the current automated methods suffer from lower detection rates and higher false-positive rates than are acceptable for obtaining valid estimates of cell numbers. Thus, at present, stereologic cell counting with manual decision for object inclusion according to unbiased stereologic counting rules remains the only adequate method for unbiased cell quantification in histologic tissue sections.

## Introduction

Stereologic cell counting has had a major impact on the field of neuroscience over the past 20 years. For example, based on several studies, until 1996 it was thought that the “benign forgetfulness” of people over 65 is due to the death of neurons in the hippocampus, a structure within the central nervous system (CNS) known to be critical to learning and memory (Wickelgren, 1996). However, stereologic cell counting has contradicted much of this work (Gallagher et al., 1996; Rapp and Gallagher, 1996; Wickelgren, 1996). Since then, the concept has emerged that age-related impairments in memory are not linked to hippocampal cell death but to specific and relatively subtle synaptic alterations in the hippocampus and prefrontal cortex (Morrison and Hof, 1997; Hof and Morrison, 2004; Morrison and Baxter, 2012). This has important consequences for developing novel treatment strategies against age-related impairments in memory (Abbott, 2012). Another important example is multiple sclerosis (MS). Scientists have thought for decades that MS is primarily characterized by chronic inflammation and loss of the myelin sheaths surrounding the axons in the CNS (Stadelmann et al., 2008). However, stereologic analyses showed in 2009 that MS is also characterized by a massive loss of motor neurons in the spinal cord (Vogt et al., 2009). Viewing MS as both inflammatory and neurodegenerative has major implications for therapy, adding CNS protection and repair to simply controlling inflammation (Luessi et al., 2012). Numerous other examples of studies highlighting the impact of stereologic cell counting in basic neuroscience and pharmaceutical and biotechnology research continue to be reported in the literature (see Part IV in Glaser et al., 2007).

**Figure 1:**
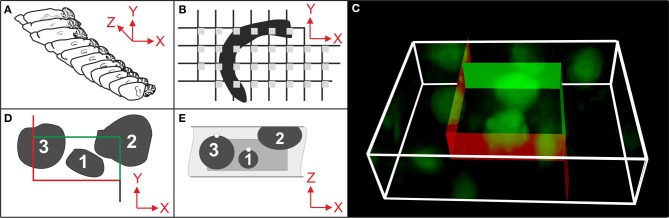
**Essential steps in stereologic cell counting, shown here for neurons in the cerebral cortex of a mouse**. **(A)** Selection of a systematically randomly sampled (SRS) series of sections through the entire region of interest (e.g., every 10th section). **(B)** Selection of a SRS series of microscopic fields-of-view (gray squares) by placing a rectangular lattice on the surface of a section, determining the positions of stereologic cell counting. **(C)** 3D rendering of a microscopic field-of-view of a 30 μm-thick section of a mouse brain showing neurons in the cerebral cortex (section processed with immunohistochemistry; anti-NeuN primary antibody; widefield fluorescent imaging performed with an Olympus BX51 microscope and 100× oil objective with N.A. = 1.4). An unbiased virtual counting space (UVCS) with red and green borders (explained in **D**,**E**) is placed within the section thickness for stereologic cell counting. **(D)** Top view (XY) of an UVCS (as shown in **C**). When viewed in 2D, the UVCS is known as an unbiased counting frame (Sterio, 1984). A cell is only counted if its profile is found either within the counting frame (cell no. 1) or intersects only the inclusion lines (green lines and cell no. 2) but not the exclusion lines (red lines and cell no. 3) of the unbiased counting frame. **(E)** Side view (XZ) on the same UVCS shown in **(D)** (dark gray) positioned within the tissue section (light gray). The tops of cells no. 1 and 3 are marked with white dots. (The top of a cell is the XYZ position of a cell within a tissue section where the cell comes into focus for the first time during microscopic imaging. A cell, such as cell no. 2, that was cut into pieces during histologic sectioning of the tissue has its top in the section above the present section.) In addition to the counting rules shown in **(D)**, cell no. 1 would be counted because its top is found within the UVCS. In contrast, cells no. 2 and no. 3 would not be counted because their top is either not found within the UVCS (cell no. 3) or not found within this tissue section (cell no. 2). Only cell no. 1 would be counted because this is the only one that fulfills all 3D stereologic counting rules shown in **(D,E)**. From all cells and the sampling probability [determined by the selected series of sections **(A)**, the grid used for positioning the UVCS **(B)** and the dimensions of the latter **(C–E)**], an unbiased estimate of the total number of cells in the CNS region of interest is calculated.

**Figure 2:**
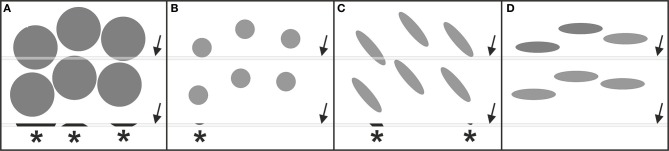
**Occurrence of systematic errors (bias) when counting cells in thin tissue sections disregarding the 3D stereologic counting rules outlined in Figure 1**. In all examples shown there is the same number and density of cells (gray elements), as well as the same spatial distribution of the cells when focusing on their midpoints. In **(A,B)** the cells differ in size, in **(B,C)** they differ in shape (but have the same volume), and in **(C,D)** they differ in their spatial orientation (but have the same shape and volume). The gray bars (arrows) represent a thin section through the tissue. Asterisks indicate cell fragments (cell profiles) that are detected in the sections. Counting the cell profiles would result in three counted cell profiles in **(A)**, one in **(B)**, two in **(C)**, and zero in **(D)**. By inspecting only the sections it is impossible to decide whether the difference in the numbers of counted cell profiles between **(A–D)** is due to different numbers of cells or due to differences in cell size, shape or orientation. This renders the results of 2D “cell counting” approaches as questionable in value for modern neuroscience.

Stereologic cell counting is performed using microscopy on histologic tissue sections (Peterson, 2004; Schmitz and Hof, 2005; Glaser et al., 2007) and has become a common laboratory method with the advent of semi-automated, computer-based microscopy systems (Stereo Investigator, MicroBrightField/MBF Bioscience, Williston, VT, USA; newCast, Visiopharm, Hoersholm, Denmark; Stereologer, Stereology Resource Center, Tampa, FL, USA). These systems integrate a 3-axes motor-driven specimen stage with a computer in order to acquire data from three-dimensional (3D) structures (Figures 1A,B,D), and implement the stereologic probes for cell counting (Figures 1C–E). However, the user must manually decide by visual inspection of the microscopic fields-of-view selected by the system whether or not a cell is counted according to the stereologic counting rules shown in Figures 1D,E. This reliance on manual inspection is the reason why stereologic cell counting has remained very labor-intensive and time-consuming to perform (weeks to months for a single study), and is the main reason why biased, non-stereologic two-dimensional (2D) “cell counting” approaches are in widespread use in basic neuroscience and pharmaceutical and biotechnology research, despite their known disadvantages and biased results (see e.g., Chapter 5 in Howard and Reed, 2010; Chapter 3 in West, 2012; and Figure 2).

Therefore, the implementation of automated 3D cell detection in stereologic cell counting would be both desirable and impactful on the field of neuroscience. (Note that automated cell detection may either be cell nuclei detection or cell cytoplasm detection, depending on the applied histologic staining/labeling. In the following text we will use the term “cell detection” for both.) The challenge of automated 3D cell detection is known in computer vision research as an object detection and segmentation problem. Object detection involves identifying locations in an image where instances of a target object appear while segmentation involves finding the entire region of the image that each object occupies. Numerous methods for automated cell detection in microscopy images have been proposed in the literature over the past 50 years (reviewed in Meijering, 2012). We performed an analysis of the theory behind many of these algorithms and found that the following methods are generally compatible with automated 3D cell detection in stereologic cell counting: (i) the technology implemented in the FARSIGHT toolkit for automated 3D cell detection (henceforth referred to as “FARSIGHT”) (Lin et al., 2007; Bjornsson et al., 2008; Al-Kofahi et al., 2010); (ii) the 3D multiple level sets (3D MLS) segmentation method (Chinta and Wasser, 2012); and (iii) the 3D Object Counter plug-in for ImageJ (Bolte and Cordelières, 2006). Additional information about the theory of cell detection in general and these methods in particular appears in the Materials and Methods section below.

FARSIGHT and the 3D MLS segmentation method were specifically developed for automated 3D cell detection in image stacks generated with confocal laser scanning microscopy (CM). Although this imaging methodology produces high spatial resolution and high signal-to-noise image data, the microscope hardware typically does not provide systematic random sampling, unless properly configured (Peterson, 2014). The concept of “systematic random sampling” is addressed in Figures 1A,B. Basically, selection of microscopic fields-of-view in a systematic random manner guarantees that all parts of the region of interest have the same chance to contribute to the sample (see e.g., Glaser et al., 2007; Howard and Reed, 2010; Mouton, 2011; West, 2012). By contrast, stereology is usually performed by imaging 25–50 μm-thick tissue sections with brightfield and widefield fluorescence microscopy (BM and FM) outfitted with a motorized stage. Compared to images obtained with CM (or BM and FM on thin sections, respectively), images obtained with BM and FM on thick tissue sections are noisier, have lower contrast and contain out-of-focus axial blur. The performance of FARSIGHT, the 3D MLS segmentation method and the 3D Object Counter plug-in for ImageJ on such tissue sections is unknown.

**Figure 3:**
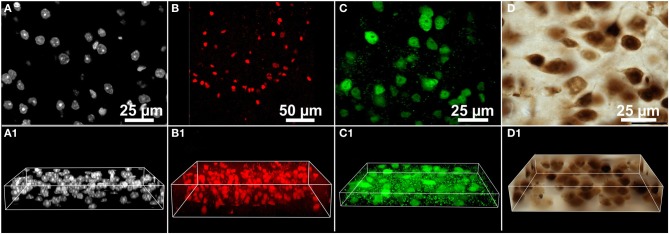
**3D microscopic images (image stacks) from the mouse brain used in benchmark tests of the performance of various methods for automated 3D cell detection**. **(A–D)** Show representative single image planes from the image stacks, and **(A1–D1)** 3D image volumes. Details are provided in the main text.

We therefore performed benchmark tests of the performance of FARSIGHT, the 3D MLS segmentation method and ImageJ's 3D Object Counter plug-in in automated 3D cell detection using a variety of 3D microscopic images (Figure 3) for which the ground truth (location and number of all nuclei/cells either fully or partially contained in the 3D images) was established by an expert observer (Susan J. Tappan) using Corel Draw software (Version 16.4.0.1280; Corel, Ottawa, ON, Canada) for manually marking nuclei/cells.

## Materials and methods

### 3D microscopic images

The benchmark tests were performed on a number of different 3D microscopic images (image stacks) from the mouse brain (Figure 3). Images stacks were acquired from various researchers and acquisition conditions. In each case, acquisition settings were determined by the original researcher. Three common experimental paradigms were represented: nuclear stain (DAPI; Figure 3A), subpopulation nuclear antibody labeling (Sox2; Figure 3B), and pan-neuronal antibody labeling (NeuN visualized with fluorescent and DAB; Figures 3C,D respectively). Care was taken to ensure that each image stack represented commonly encountered variations in cell size, number, and density. For each image stack the following information is provided in Table 1: brain region, section thickness, staining/labeling, microscope, camera, illumination mode, objective lens used, numerical aperture of the objective lens, pixels per image plane, lateral pixel spacing, distance between the image planes, total number of image planes, provider of the section, ground truth. All image data utilized for these experiments are available to view at the following location (www.biolucida.net/Automated3DCellDetection).

**Table 1:** **Details of the 3D microscopic images (shown in Figure 3) used in benchmark tests of the performance of various methods for automated 3D cell detection**.

**Figure**	**3A**	**3B**	**3C**	**3D**
Brain region	Cerebral cortex	Hippocampus	Cerebral cortex	Striatum
Section thickness	30μm	50μm	30μm	30μm
Staining/labeling	Histochemistry (DAPI stain)	Immuno-histochemistry (anti-Sox2 primary antibody; visualization of antibody binding with CY3)	Immuno-histochemistry (anti-NeuN primary antibody; visualization of antibody binding with Alexa-488	Immuno-histochemistry (anti-NeuN primary antibody; visualization of antibody binding with DAB
Microscope	Zeiss M2 Apotome	Olympus FluoView FVX confocal	Olympus BX51	Olympus BX51
Camera	MRM (Zeiss, Jena, Germany)	n/a (photomultiplier)	Ocra R2 (Hamamatsu, Hamamatsu City, Japan)	2000R (QImaging, Surrey, BC, Canada)
Illumination mode	Fluorescence	Confocal fluorescence	Disc spinning unit	Brightfield
Objective lens used	63× oil	20×	60× oil	100× oil
Numerical aperture of the objective lens	1.4	0.7	1.4	1.4
Pixels per image plane	1388 × 1040	512 × 512	1344 × 1024	1600 × 1200
Lateral pixel spacing	X: 0.102134 Y: 0.101507μm/pixel	X: 0.46056 Y: 0.46056μm/pixel	X: 0.102134 Y: 0.101507μm/pixel	X: 0.075173 Y: 0.074300μm/pixel
Distance between the image planes	0.5μm	1μm	0.5μm	0.5μm
Total number of image planes	45	51	26	45
Provider of the section	a	D.A.P.	C.S.	b
Ground truth	154 nuclei	246 nuclei	53 neurons	58 neurons

*Notes: a, Drs Lissa Ventura-Antunes and Suzana Herculano-Houzel (Lab. Comp. Neuroanat.; Univ. Federal do Rio de Janeiro, Brazil); b, CHDI Foundation (New York, NY)*.

### Manual validation for establishing ground truth

When manually marking cells, characteristics of the target population with respect to expression pattern of the label, object (cell) size, and the amount of tissue shrinkage observed are factored in. In the present experiment, objects were considered cells if the label was observed in the nucleus (having an average diameter of 4–5 μm in XY). NeuN labeling was also visible in the cytoplasm (Figures 3C,D). Cells were identified on the basis of positive labeling, without regard to staining intensity. Although size considerations are critical in discriminating between neuronal and non-neuronal cells with a non-specific label such as DAPI, no distinction was made in the present experiment. Each image stack was loaded into Corel Draw software. Image adjustments were made as necessary to optimize the visualization of the target population (cell nucleus or cytoplasm). A marker was used to identify the centroid of each cell wholly contained within the image volume, while a second marker was used to identify the centroid of cells that intersected the image boundary. In this manner, all cells were marked. The centroid of the cell was identified by the median image plane in which the cell appeared. After cells were marked, the image volume was inspected to ensure that no cells were missed. Each image stack required between 40–180 min to be marked manually; the image stack designated in Figure 3B required the greatest amount of time (180 min) to validate manually. The XYZ coordinates of all markers were noted in an Excel spreadsheet.

### 3D cell detection methods

Cell detection is a specific application of the larger class of object detection and segmentation problems. The computer vision and image processing literature contains many algorithms for 2D and 3D cell detection. Meijering provides a recent overview of the variety of approaches used to address this problem (Meijering, 2012). The common approach for all of these techniques is to establish a model of what a cell looks like and search for parts of an image that match the model.

The 3D Object Counter plug-in for ImageJ uses the simplest model of cell appearance: it uses a single intensity threshold to divide an image into cell (foreground) and non-cell (background) regions. The algorithm then employs connected components—a classic computer vision algorithm—to designate each connected region of the foreground as a separate cell (Shapiro and Stockman, 2001). Because of its simple model of cell appearance, this algorithm suffers from several problems when used outside of very tightly controlled environments. First, in the case of cell segmentation, variations in staining intensity within cells make it difficult to select a single intensity level that separates all cells from the background. Second, cells that are close together may appear within the same foreground connected component and will not be recognized as separate objects. Despite these known problems, we selected this algorithm for inclusion in this study because of its wide availability as one of the freely available plug-ins for ImageJ.

More sophisticated segmentation algorithms use local image information to separate objects from the background and to separate neighboring foreground objects from each other. The 3D MLS algorithm is one of several cell segmentation techniques based on level-set methods, which model the boundary of an object as the 0-level of an equation (Dzyubachyk et al., 2008; Chinta and Wasser, 2012; Qi et al., 2012). The shape of the boundary is controlled by modifying the equation based on image content and model constraints. One advantage of level-set methods is that they provide a natural way to handle splitting and merging object contours. The 3D MLS algorithm uses k-means clustering (Jain, 1988) followed by expectation and maximization (Zhao and Murphy, 2007) to separate the foreground from the background. A level-set function is established for each connected component of the initial segmentation and level-set evolution proceeds in two stages. First, regions are eroded, with splitting, to find a set of seed points at the centroid of each detected cell. Second, regions are grown to determine the boundaries of each cell. While there are other similar level-set-based methods for cell segmentation, we selected this variant because it is the most recent and the authors provide a reference Windows executable application to apply the algorithm to any data set.

The FARSIGHT “nuclei segmentation method” is the latest in a line of methods explored by the developers of the FARSIGHT toolkit (Lin et al., 2003, 2007; Bjornsson et al., 2008; Padfield et al., 2008; Al-Kofahi et al., 2010). Note that the FARSIGHT toolkit itself is not a software package but a collection of modules for quantitative analysis of tissue sections. In order to use the toolkit one has to choose the right set of modules and stitch them together using a scripting language such as Python. However, the toolkit comes packaged with several such components stitched together for performing specific analyses. We selected the latest nuclei segmentation approach described from this group (Al-Kofahi et al., 2010). The FARSIGHT algorithm uses a four-step approach. Initial foreground and background separation is based on graph cutting, which considers local image information at each pixel as well as the classification of neighboring pixels (Boykov et al., 2001). Cell seed point detection is based on the response to a multiple scale Laplacian of Gaussian filter, a detector that highlights regions of local image contrast of different sizes (Lindeberg, 1998). Region growing from the seed points is based on local-maximum clustering, which suppresses faint seed points detected close to prominent seed points (Wu et al., 2004). Finally, the boundaries of connected objects are modified using alpha-expansions for multiple label graph cuts (Boykov et al., 2001).

Besides ImageJ, 3D MLS, and FARSIGHT, we considered other recently described 3D cell segmentation algorithms, but ultimately excluded them from evaluation for the following reasons. Li et al. (2007) used image gradient flow tracking to find the centroids of nuclei in 3D followed by locally-adaptive thresholding. The dependence on image gradients to find the centroids of cells limits this method to cells that do not contain texture. The locally-adaptive thresholding approach does not separate nearby cells. Bashar et al. (2012) employed multiple scale smoothing filters followed by non-maximum suppression and distance-based clustering to identify nuclei centroids. This approach is similar to the early stages of the FARSIGHT algorithm, and can therefore be considered a subset. Lou et al. (2012) used a graph-cut segmentation approach similar to FARSIGHT's approach and the authors did not provide a reference implementation. Wählby et al. (2004) used a watershed-based segmentation with subsequent merging and splitting steps based on the strength of image gradients. The authors provided a single example of this method applied to 3D cell detection, but they did not provide a reference implementation.

There are two classes of cell detection algorithms that operate in 2D. The first of these were developed for working with very thin tissue sections that are only examined with 2D images and the 3D shape of the cells is completely disregarded (Baggett et al., 2005; Yu et al., 2010; Wienert et al., 2012). Such methods are inappropriate for dealing with the thick tissue sections and 3D images used in stereology. The second class of 2D approaches first seek to identify cell profiles in 2D image slices and then aggregate information found in multiple planes to build a full 3D segmentation (Indhumathi et al., 2011; Leung et al., 2011). The aggregation methods are largely based on heuristics (e.g., linearity of centroids and analysis of convexity) rather than complete models of cell appearance. Moreover, there is larger variability in shape, size and appearance of 2D cell profiles than there is for 3D cells. For example, some profiles will be much smaller than others, some profiles may include a nucleus while others do not, and some profiles may be split into two regions within the same plane. Decomposing the segmentation problem to 2D profile detection and then aggregating into a 3D segmentation introduces more problems than it solves. Put simply, because stereology operates on 3D images, the principled approach is to use a 3D segmentation algorithm.

### Benchmark tests

The evaluation was performed on a desktop computer equipped with an Intel Quad Core 3.6 GHz processor, 64 GB RAM, and the Windows 7 64-bit operating system. Of the evaluated methods, only FARSIGHT makes use of more than one processor core.

FARSIGHT version 0.4.5 was downloaded from the FARSIGHT website (http://www.farsight-toolkit.org/wiki/Main_Page). Default operation involved launching the Nucleus Editing Tool (NucleusEditor.exe) and selecting Tools/Segment Nuclei. In this mode, the application uses an adaptive para-meter selection algorithm. Manual operation involved running the segment_nuclei.exe program from the Windows command line and providing algorithm parameters through a configuration file as detailed on the FARSIGHT website (http://www.farsight-toolkit.org/wiki/Nuclear_Segmentation). Parameters were selected according to the descriptions of the parameters provided in the FARSIGHT documentation. Several iterations of empirical parameter tuning were completed until the segmentation result appeared to capture the greatest number of cells with the least amount of oversegmentation.

The 3D Segmentation Tool for Cell Nuclei Detection (referred to as 3D MLS) version 1.02 was downloaded from its website (http://web.bii.a-star.edu.sg/archive/MLSCellNucleiDetection/). Images were converted from TIFF format to Image Cytometry Standard (ICS) format using Huygens Essential software (Scientific Volume Imaging B.V., Hilversum, The Netherlands). Default operation involved selecting Tools/3D Cell Nuclei Segmentation and accepting all default parameters. Manual operation involved selecting algorithm parameters according to instructions provided in the online supplemental material for a study by Chinta and Wasser (2012). Several iterations of empirical parameter tuning were completed until the segmentation result appeared to capture the greatest number of cells with the least amount of oversegmentation and undersegmentation.

ImageJ version 1.48d was installed from the Fiji is Just ImageJ (Fiji) distribution website (http://fiji.sc/Fiji) which includes the 3D Object Counter version 2.0 plug-in (http://rsbweb.nih.gov/ij/plugins/track/objects.html). Manual operation involved selecting Analyze/3D Objects Counter and adjusting the threshold level to include as many visible cells as possible while separating nearby cell regions. The size filter was used to exclude objects that are much smaller than a typical cell, i.e., 1000 pixels for the data sets in Figures 3A,C,D (5.2, 5.2, and 2.8 μm^3^, respectively) and 25 pixels for the data set in Figure 3B (5.3 μm^3^). Smoothing operation involved first convolving the image data with a 3D Gaussian filter (Process/Filters/Gaussian Blur 3D) with standard deviations in pixels of {σ_*x*_ = 2, σ_*y*_ = 2, σ_*z*_ = 1}. The purpose of this smoothing operation was to reduce the effect of camera noise on the segmentation. Accordingly, the scale of the Gaussian operator was independent of the optical resolution.

All of the evaluated segmentation programs expect as input a single channel 3D image in which the target objects (cell nuclei or cytoplasm) appear bright on a dark background, as occurs in fluorescent microscopic imaging. For fluorescent microscopic images, the single channel that targeted the nuclear (DAPI or Sox-2) or cytoplasmic label (NeuN) was saved as a separate 3D image file and loaded into the respective segmentation programs.

**Figure 4:**
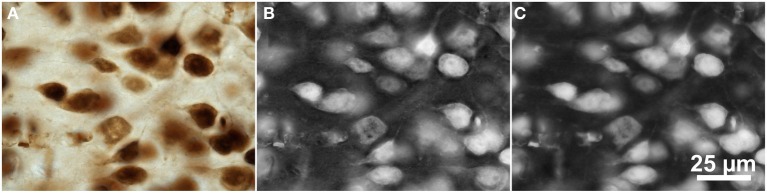
**Color space manipulations of the brightfield microscopic image from Figure 3D (mouse cerebral cortex, anti-NeuN primary antibody; visualization of antibody binding with DAB, brightfield microscopy)**. **(A)** The original RGB image. **(B)** The inverted red channel. **(C)** The red chromaticity channel from an Lrg color space conversion. Scale in **(C)** for **(A–C)**.

Two approaches were used to extract single channel images from the brightfield microscopic images of NeuN-labeled tissue (Figure 3D) in which the cells appear bright against a dark background, as shown in Figure 4. The original image data was acquired with a color camera and saved in the RGB color space (e.g., Figure 4A). In these images, the red channel contained the highest contrast and the cell regions had a darker red level than the background. The first approach therefore involved inverting the red channel and saving it as a separate 3D image file for segmentation (Figure 4B). The other approach involved converting the original RGB color image to the Lrg color ratio space, which separates intensity (luminance) from color (chromaticity) (Szeliski, 2011). The red chromaticity value for a single pixel was computed as *r = R / (R + G + B*), where *R*, *G*, and *B* are the original pixel's red, green, and blue values, respectively. Because this color conversion operated on each pixel independently, it affected only the contrast of the image and not the image resolution. The cell regions in this red chromaticity channel appear brighter than the background, so the second approach involved saving the red chromaticity channel as a separate 3D image file for segmentation (Figure 4C).

All of the evaluated segmentation programs produce as output a labeled 3D image file of the same size as the input image in which the pixels belonging to each segmented object are indicated with a unique value. We computed from the labeled 3D images the locations of the region centroids for use in visualization and analysis. Let *k* be a unique region label and Ω_*k*_ be the set of pixels in a 3D image with this label. The centroid of this region is provided by the equation:

$$\begin{array}{l} C_{k}=\left(\frac{\sum_{x,y,z\in \Omega_{\text{k}}}x}{\left|\Omega_{k}\right|},\;\frac{\sum_{x,y,z\in \Omega_{\text{k}}}y}{\left|\Omega_{k}\right|},\frac{\sum_{x,y,z\in \Omega_{\text{k}}}z}{\left|\Omega_{k}\right|}\right),\\ \;|\Omega_{k}|=\sum_{x,y,z\in \Omega_{k}}1. \end{array}$$

**Figure 5:**
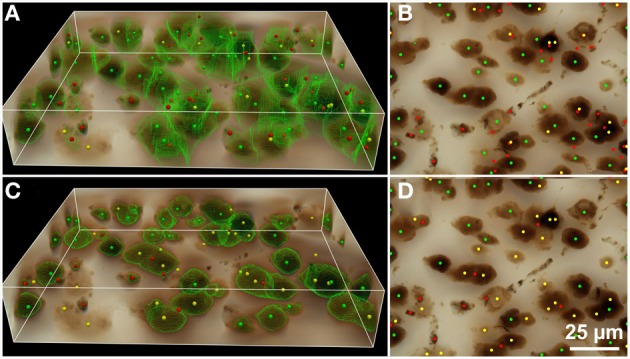
**Results of automated 3D cell detection on the 3D microscopic image from Figures 3D,D1 (mouse cerebral cortex, anti-NeuN primary antibody; visualization of antibody binding with DAB, brightfield microscopy) using FARSIGHT (Al-Kofahi et al., 2010) (A,B) and the 3D Object Counter plug-in for ImageJ (Bolte and Cordelières, 2006) (C,D)**. The 3D MLS segmentation method failed to execute on this image data for unknown reasons (the software crashed during initial foreground/background separation). **(A,C)** Show 3D reconstructions of the image stack overlaid with green contours that define the physical extent of a detected object in each image plane; **(B,D)** show minimum intensity projections of the image stack. Green dots indicate where there was a match between a ground truth cell and an automatically detected object (true-positive); yellow dots indicate where there was a ground truth cell that was not detected automatically (false-negative); red dots indicate where the detection algorithm found an object that is not a cell (false-positive). Scale in **(D)** for **(B,D)**.

**Figure 6:**
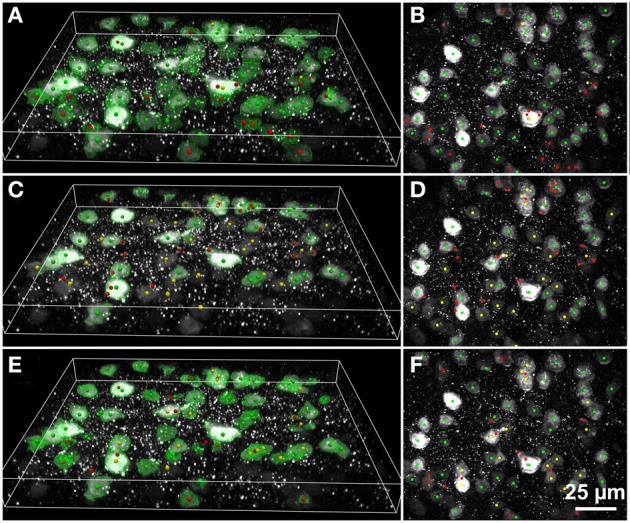
**Results of automated 3D cell detection on the 3D microscopic image from Figures 3C,C1 (mouse cerebral cortex, anti-NeuN primary antibody; visualization of antibody binding with Alexa-488, confocal spinning disc microscopy) using FARSIGHT (Al-Kofahi et al., 2010) (A,B), the 3D MLS segmentation method (Chinta and Wasser, 2012) (C,D) and the 3D Object Counter plug-in for ImageJ (Bolte and Cordelières, 2006) (E,F)**. **(A,C,E)** Show 3D reconstructions of the image stack overlaid with green contours that define the physical extent of a detected object in each image plane; **(B,D,F)** show minimum intensity projections of the image stack. Green dots indicate where there was a match between a ground truth cell and an automatically detected object (true-positive); yellow dots indicate where there was a ground truth cell that was not detected automatically (false-negative); red dots indicate where the detection algorithm found an object that is not a cell (false-positive). Scale in **(F)** for **(B,D,F)**.

**Figure 7:**
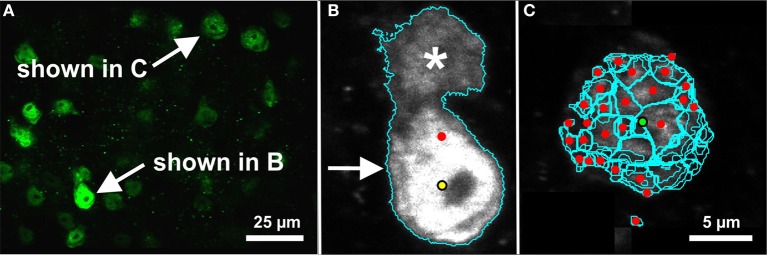
**Evaluation of segmentation errors**. Note that these images contain representative image planes and segmentation contours, but the image data and segmentations are 3D. Both examples are shown on a certain image plane of the microscopic 3D image shown in Figure 3C (mouse cerebral cortex, anti-NeuN primary antibody; visualization of antibody binding with Alexa-488, fluorescence microscopy). **(A)** The image plane in which the segmentation errors occurred. The corresponding cells are indicated. **(B)** The automated segmentation algorithm (3D Object Counter plug-in for ImageJ; Bolte and Cordelières, 2006) identified an object (cyan contour; arrow) in the same region as a ground-truth cell (yellow dot). However, the boundary of the automated segmentation included an adjacent cell (asterisk), which displaced the centroid of the automatically segmented object (red dot). This situation was properly classified as a segmentation error. **(C)** The automated segmentation algorithm (FARSIGHT; Al-Kofahi et al., 2010) oversegmented a cell. Although the centroid of one of these regions was close enough to the ground truth position (green dot), the remaining regions were all classified as errors (red dots). Such oversegmentations led to true-positive and false-positive rates near 100%. Scale in **(C)** for **(B,C)**.

In addition, we found the region boundaries in each *XY* image plane for use in visualizations, such as Figures 5–7. The cell centroid and boundary data were saved to a data file for further analysis.

Evaluation required determining where an automated segmentation algorithm identified an object that was also identified in the ground truth data. Our analysis was based on the assumption that if the two methods have identified the same object, the centroid of that object will be in approximately the same location. We evaluated each automated segmentation result against the ground truth data by matching cell centroids identified by both the automated method and the expert observer. This was first done for all cells in the ground truth data, and afterwards only for those cells in ground truth that were completely contained within the image boundary. The ground truth and automated cell centroid locations were read in from data files. A search region centered on each ground truth cell centroid was inspected to find the nearest automatically detected cell centroid. If a centroid was found within the search region, the ground truth cell centroid was marked as a “true-positive” and the automatically detected centroid was removed from further consideration. Otherwise, the ground truth cell centroid was marked as a “false-negative.” After all ground truth cells were considered, remaining unmarked automatically detected centroids were marked as “false-positives.” All cells contained in the datasets shown in Figure 3 were analyzed by the automated 3D cell detection methods and the expert observer, regardless of whether they were completely contained within the image stack or touched the boundary of the image stack.

The search region defined the allowed error tolerance used to match a manually identified cell centroid with an automatically detected cell centroid. We chose a cylindrical search area to approximate the appearance of a spherical cell imaged with the point spread function of the microscope. The decreased axial resolution available in light microscopy images means there is more precision determining the XY coordinates of a cell centroid than the Z coordinate. We therefore used a lateral (XY) tolerance radius of 3 μm based on the lower end of observed cell nucleus radii. We selected the axial (Z) tolerance based on a fraction of the number of image planes for which most cells are in focus—an axial tolerance of 1.5 μm was used for the data sets in Figures 3A,C,D and 2.0 μm was used for the data set in Figure 3B owing to the lower numerical aperture of the objective lens used when acquiring this data set.

True-positives were objects identified by both the ground truth and automated segmentation. False-negatives were objects identified in the ground truth data but not the automated segmentation. False-positives were objects identified by the automated segmentation but not the ground truth. True-positive and false-positive rates were computed as follows:

$$R_{tp}=\frac{N_{tp}}{N_{gt}};\;R_{fp}=\frac{N_{fp}}{N_{s}}$$

with *R_tp_* the true-positive rate, *N_tp_* the number of true-positives, *N_gt_* the number of ground truth, *R_fp_* the false-positive rate, *N_fp_* the number of false-positives, and *N_s_* the number of automatically segmented objects. A successful segmentation algorithm simultaneously attains a high true-positive rate and a low false-positive rate.

In a final experiment, we combined for each data set shown in Figure 3 the best performing mode of FARSIGHT (determined in Table 2) with the best performing modes of either the 3D MLS segmentation method or ImageJ's 3D Object Counter plug-in, respectively. Specifically, we established the result of using both automated segmentation methods simultaneously by finding the unions of the true-positive and false-positive object centroids identified by the two methods.

**Table 2:** **Results of benchmark tests of the performance of various methods for automated 3D cell detection on the 3D microscopic images shown in Figure 3**.

**P**	**Method**	**Mode**	***N_gt_***	***N_gti_***	***N_s_***	***N_tp_***	***N_fn_***	***N_fp_***	***R_tp_***	***R_tpi_***	***R_tpb_***	***R_fp_***	**Note**
A	F	D	155	56	1344	153	2	1191	0.99	1.00	0.98	0.89	
	F	M	155	56	138	133	22	5	0.86	0.93	0.82	0.04	
	3DMLS	D	155	56	219	117	38	102	0.76	0.73	0.77	0.47	
	3DMLS	M	155	56	130	114	41	16	0.74	0.66	0.78	0.12	
	ImageJ	M	155	56	91	54	101	37	0.35	0.34	0.35	0.41	
	ImageJ	S	155	56	96	54	95	36	0.39	0.36	0.40	0.38	
B	F	D	246	162	298	204	42	94	0.83	0.83	0.82	0.32	
	F	M	246	162									a
	3DMLS	D	246	162	202	167	79	35	0.68	0.69	0.67	0.17	
	3DMLS	M	246	162	162	130	116	32	0.53	0.57	0.45	0.20	
	ImageJ	M	246	162	313	159	87	154	0.65	0.69	0.57	0.49	
	ImageJ	S	246	162	122	60	186	62	0.24	0.17	0.38	0.51	
C	F	D	60	34	2349	59	1	2290	0.98	1.00	0.96	0.98	
	F	M	60	34	69	56	4	13	0.93	0.97	0.89	0.19	
	3DMLS	D	60	34	51	30	30	21	0.50	0.50	0.50	0.41	
	3DMLS	M	60	34	53	30	30	23	0.50	0.50	0.50	0.43	
	ImageJ	M	60	34	52	40	20	12	0.67	0.59	0.77	0.23	
	ImageJ	S	60	34	59	35	25	24	0.58	0.50	0.69	0.41	
D	F	D	58	35	567	32	26	535	0.55	0.54	0.57	0.94	
	F	M	58	35	120	22	36	98	0.38	0.43	0.30	0.82	
	F	R	58	35	76	33	25	43	0.57	0.54	0.61	0.57	
	3DMLS	D	58	35									b
	3DMLS	R	58	35									b
	ImageJ	M	58	35	41	27	31	14	0.47	0.31	0.70	0.34	
	ImageJ	S	58	35	37	29	29	8	0.50	0.40	0.65	0.22	
	ImageJ	R	58	35	39	28	30	11	0.48	0.34	0.70	0.28	
	ImageJ	R-S	58	35	37	29	29	8	0.50	0.37	0.70	0.22	

*P, panel in Figure 3. F, the functionality implemented in FARSIGHT for 3D automated cell detection (Lin et al., 2007; Bjornsson et al., 2008; Al-Kofahi et al., 2010); 3DMLS, 3D MLS (multiple level sets) segmentation method (Chinta and Wasser, 2012); ImageJ, 3D Object Counter plug-in for ImageJ (Bolte and Cordelières, 2006). D, default mode; M, manual mode; S, smoothed mode (images pre-processed with a 3D Gaussian filter); R, conversion of the 3D image into the Lrg color space, which separates intensity (luminance) from color (chromaticity), and running the segmentation on the red channel. N_gt_, number of cells in ground truth; N_gti_, number of cells in ground truth completely contained within the image boundary; N_s_, number of objects detected by the software; N_tp_, number of matching cells (true-positives); N_fn_, number of missed cells (false-negatives); N_fp_, number of false-positives; R_tp_, rate of true-positives; R_tpi_, rate of true-positives for cells completely contained within the image boundary; R_tpb_ rate of true-positives for cells that touch at least one image boundary; R_fp_, rate of false-positives. Notes: a, no combination of manually selected parameters produced any segmented objects; b, software crashed for unknown reasons during initial foreground/background separation*.

## Results

The results of the benchmark tests are summarized in Tables 2, 3. FARSIGHT performed the best compared to the other methods in all image data sets shown in Figure 3, with true-positive rates from 38 to 99%. However, the default mode of FARSIGHT—which automatically selects algorithm parameters—worked successfully only on the data set shown in Figure 3B (true-positive rate 83%, false-positive rate 32%). On the other data sets, the default mode of FARSIGHT yielded false-positive rates from 89 to 98%, indicating complete segmentation failures that should be disregarded. Manually selecting algorithm parameters improved the performance of FARSIGHT on the datasets shown in Figure 3A (small decrease in the true-positive rate from 99 to 86%, but large decrease in the false-positive rate from 89 to 4%) and Figure 3C (small decrease in the true-positive rate from 98 to 93%, but large decrease in the false-positive rate from 98 to 19%). However, this method is still prone to oversegmentation and erroneous detections (false-positive rates up to 82% found on the dataset shown in Figure 3D despite manually selecting algorithm parameters). Importantly, manual parameter selection is not possible through FARSIGHT's graphical user interface (GUI).

**Table 3:** **Results of benchmark tests of the combined performance of various methods for automated 3D cell detection on the 3D microscopic images shown in Figure 3**.

***P***	***M1***	***M2***	***GT***	***M1-R_tp_***	***M1/2-R_tp_***	***M1-R_fp_***	***M1/2-R_fp_***
A	F–M	X–M	155	0.86	0.91	0.036	0.11
	F–M	J–M	155	0.86	0.87	0.036	0.22
B	F–D	X–D	246	0.83	0.87	0.32	0.33
	F–D	J–M	246	0.83	0.92	0.32	0.50
C	F–M	X–D	60	0.93	0.95	0.19	0.35
	F–M	J–M	60	0.93	0.95	0.19	0.27
D	F–R	J–R	58	0.57	0.74	0.57	0.53

*P, panel in Figure 3. M1, first selected 3D cell detection method. M2, second selected 3D cell detection method. F, the functionality implemented in FARSIGHT for automated 3D cell detection (Lin et al., 2007; Bjornsson et al., 2008; Al-Kofahi et al., 2010); X, 3D MLS (multiple level sets) segmentation method (Chinta and Wasser, 2012); J, 3D Object Counter plug-in for ImageJ (Bolte and Cordelières, 2006); M, manual mode; D, default mode; R, conversion of the 3D image into the Lrg color space, which separates intensity (luminance) from color (chromaticity), and running the segmentation on the red channel. M1-R_tp_, rate of true-positives when using the first selected method; M1/2-R_tp_, rate of true-positives when using both the first and second selected method and combining the results; M1-R_fp_, rate of false-positives when using the first selected method; M1/2-R_fp_, rate of false-positives when using both the first and second selected method and combining the results*.

FARSIGHT detected the brightfield image data (Figure 3D) best when the image was converted into the Lrg color space, which separates intensity (luminance) from color (chromaticity), and the segmentation was run on the red chromaticity channel (Figure 5), obtaining a true-positive rate of 57% and a false-positive rate of 57%. Note that because this color conversion operated on each pixel independently, it affected only the contrast of the image and not the image resolution. However, ImageJ's 3D Object Counter plug-in obtained a slightly worse true-positive rate (50%) with a much better false-positive rate (22%) for this dataset when operating on images pre-processed with a 3D Gaussian filter. Unfortunately, the 3D MLS segmentation method failed to execute on the brightfield image data shown in Figure 3D. No combination of parameters could be found for which the software passed the initial foreground/background separation stage. Because no source code is available for this application, we could not determine what caused this issue.

The 3D MLS segmentation method performed better than ImageJ's 3D Object Counter plug-in on the data set shown in Figure 3B (immunofluorescent nuclear labeling), whereas it was the opposite for the data set shown in Figure 3C (immunofluorescent cytoplasmic labeling; Figure 6). Smoothing was found to significantly reduce the amount of time required for the ImageJ segmentation, but this did not significantly change the results.

In almost all cases, the true-positive rate for the cells that were completely contained within the image boundary (*R_tpi_*) was no better and usually slightly worse than the true-positive rate for all cells (*R_tp_*) (Table 2). In other words, the interior cells were not always detected and the cells that touched at least one image boundary were not always missed. The most significant difference occurred in the case of FARSIGHT on the dataset shown in Figure 3A, where *R_tpi_* = 93% and *R_tp_* = 86%. Note, however, that the automated algorithms do not provide a valid distinction between cells that are completely contained within the image boundary and cells that touch at least one image boundary because the automated segmentations often encompass a much greater volume than the cells themselves. For example, of the 56 manually identified cells that were completely contained within the image boundary in the data set shown in Figure 3A, FARSIGHT identified only 12 cells (22%) with segmentations that were also completely contained within the image boundary.

We observed two potential problems with the automated analysis. First, in some cases a false-negative and false-positive appeared in close proximity. While it is possible that the expert observer and segmentation algorithm were identifying features of the same object, the extents of the object determined by the segmentation algorithm displaced the centroid of the object, so this was properly considered a segmentation error (Figure 7A). Second, it was possible for the segmentation algorithm to compute incorrect extents of an object but the centroid happened to coincide with a ground truth point, leading to an improperly classified true-positive. While we did not account for this, visual inspection determined that this was a rare occurrence outside of completely erroneous oversegmentations, which led to true-positive and false-positive rates close to 100% (Figure 7B).

Combining several of the automated 3D cell detection methods using the union of their detected objects resulted in improved true-positive rates, but also in increased false-positive rates when compared to the results of the individual methods. The greatest improvement in the rate of true-positives was found when combining FARSIGHT with ImageJ's 3D Object Counter plug-in for the analysis of the brightfield image data shown in Figure 3D [the true-positive rate increased from 57% (FARSIGHT) and 50% (ImageJ) to 74%]. We also investigated using the intersection of results provided by multiple automated 3D cell detection methods (i.e., an object must be detected by both automated algorithms to be considered a cell). This predictably resulted in low true-positive rates (33–68%) and low false-positive rates (3.3–20%).

## Discussion

Unbiased stereologic cell counting techniques are currently the most reliable methods to obtain valid estimates of cell numbers in histologic tissue specimens (Glaser et al., 2007; Howard and Reed, 2010; Mouton, 2011; West, 2012). In recent years, computer-integrated microscopy has enabled the rapid collection of large amounts of image data. There has been a consequent increase in the efforts to provide automated microscopic image analysis, but this has remained a bottleneck. The integration of automated cell detection techniques is an obvious goal for increasing the throughput of stereologic cell counting. However, several factors still stand in the way.

First, current automated cell detection methods are not accurate enough. In this regard it is important to note that generally accepted criteria are not available to establish a minimum benchmark that automated techniques would need to obtain. For example, one could request that automated techniques consistently obtain a true-positive rate greater than 90% and false-positive rate lower than 10%. This may be sufficient for certain but not all biological questions. The data in Tables 2, 3 demonstrate that of the current automated cell detection techniques, FARSIGHT performed best with true-positive rates between 38 and 99%, and false-positive rates between 3.6 and 82%. Specifically, best performance was obtained in the present investigation using FARSIGHT on the datasets shown in Figures 3A,C (both cerebral cortex) with manual selection of algorithm parameters, resulting in true-positive rates of respectively 86 and 93% and false-positive rates of respectively 4 and 19%. This level of performance remains poor compared to desired rates of, e.g., consistently greater than 90% for true-positive rates and consistently lower than 10% for false-positive rates. On the datasets shown in Figure 3B (hippocampus) and Figure 3D (striatum) the performance of FARSIGHT was worse than on the data sets shown in Figures 3A,C (both neocortex). This may indicate that the performance of FARSIGHT depends on the cell density in the investigated tissue (which is higher in hippocampus and striatum than in the neocortex of mice). It should also be noted that because the three automated methods find a different set of ground truth cells, the performance of FARSIGHT could be improved by incorporating the results from 3D MLS segmentation and ImageJ's 3D Object Counter plug-in. However, the simple union of results we obtained in the present study does not offer a sufficient improvement. This procedure increased true-positive rates but also false-positive rates (as shown in Table 3). An interesting avenue of future research would investigate machine-learning algorithms to combine the results from multiple automated methods.

Second, the current methods are not robust enough to account for the diversity of cell appearance encountered in tissue. Neuroanatomical areas are often not homogenously distributed in terms of cell packing density and cell clustering. This means that automated cell detection parameters tuned on one image stack may not be directly transferrable to another image stack from the same study. When manually analyzing histologic tissue sections, knowledge of the expression pattern of the label (e.g., antigen) used to visualize the population of interest, the characteristic size and shape ranges of that cell population, and histologic preparation protocols are considered. It is easy for a human to interpret that a labeled object that appears only on the top one or two images planes of an image volume to represent a bisected cell, since we have an expectation (knowledge) that cells will be, for example, approximately 10 micrometers in diameter. Different cell types have different size ranges, as well as shapes (pear or tear shaped, ovoid, spherical, or pyramidal), which inform a scientist when manually identifying cells. Histologic preparation considerations also factor in, because some degree of tissue shrinkage will occur and this changes the size of the cells (but not their presence) in *Z*. All this information is utilized by the observer who will disregard groups of pixels as non-specific labeling, or too small or too large to be a single cell. While boundary exclusion criteria can be employed to restrict consideration of objects that are not fully contained within the image volume, this can require more image stacks to be analyzed since using the top of the labeled object (which a manual counter will typically choose as a counting criterion) is less restrictive than the centroid—as the centroid criterion effectively means that one is excluding objects from consideration if the top/bottom of a cell is cut off within the image volume.

In a very recent study another 3D cell detection method was presented that was described as “robust to the broad diversity of shape, size and density of the neurons in a mouse brain” and “allows locating the neurons across different brain areas without human intervention” (Quan et al., 2013). These authors applied their method on tissue sections of brains from Thy-1-eGFP-H and Thy1-YFP-M transgenic fluorescence mice, and reported an overall true-positive rate of 88%, and a false-positive rate of 8% compared with manually detected positions of neurons as the ground truth. We could not test the method of Quan et al. (2013) on the image datasets shown in Figure 3 because there is no reference implementation available for this method. However, it should be noted that in the brains from Thy-1-eGFP-H and Thy1-YFP-M transgenic fluorescence mice, only a subset of approximately 10% of all neurons in the cerebral cortex express eGFP (Feng et al., 2000), resulting in a much lower cell density than shown in Figure 3. Accordingly, one can hypothesize that the method of Quan et al. (2013) would result in lower true-positive rates and higher false-positive rates when applied to image sets from specimens typically used by researchers for stereologic cell counting.

Overall, the automated cell detection literature is dense with single dataset papers, in which a method is shown to work on a limited and homogeneous set of image data and not evaluated against other techniques or other image data. The situation is recognized by the research community: Meijering (2012) recently commented that “the technical literature is full of alleged great methods, which were claimed to beat all previous methods for a given application, but subsequently disappeared into oblivion because no one was able to use or reproduce them” (Meijering, 2012). In this regard Chinta and Wasser (2012) stated that FARSIGHT does not require any parameter adjustment, but as demonstrated in the present study, this statement is in fact incorrect. Manually selecting algorithm parameters did improve the performance of FARSIGHT, as shown in Table 2. In the image data set investigated by Chinta and Wasser (2012)—live *Drosophila* embryos expressing histone-2Av-green fluorescence protein imaged with a confocal laser scanning inverted microscope (Zeiss 5 Live; Carl Zeiss, Jena, Germany) and a 63×/1.4 oil DIC Plan-Apochromat objective—the use of FARSIGHT resulted in slight oversegmentation, supposedly related to increased seed detection due to spatially distinct clusters of condensed chromatin (Chinta and Wasser, 2012). In contrast, FARSIGHT performed better than Chinta and Wasser's (2012) 3D MLS segmentation method in all image data sets investigated in the present study (c.f. Table 2).

Meijering (2012) has proposed a two-step process to improve the unsatisfactory situation addressed in the present study. First, he pointed out that microscopic imaging, biological experimentation, and computer hardware development all underwent major revolutions in the past decades. In contrast, most cell image analysis methods are still based on textbook ingredients (Wu et al., 2008) that were developed during the early days of cell image analysis (Meijering, 2012). Accordingly, recently proposed methods are rarely based on a single new concept, but are often merely new combinations of earlier approaches, tailored to a specific application (Meijering, 2012). Meijering (2012) proposed to design novel methods for cell detection in such a way that they are sufficiently generic to be easily trainable for a wide range of applications while consistently achieving high sensitivity and specificity in each case. Second, Meijering (2012) pointed out that the organization of open challenges based on standardized test data and criteria should suppress the practice that certain methods may be easily abused by others to “prove” superiority of their own methods, and can be expected to further accelerate progress in the field in the near future. Making the image data utilized for the experiments in the present study available to the scientific community (at www.biolucida.net/Automated3DCellDetection) may serve as a first step to achieve this goal.

Finally, it should be mentioned that recently another method was developed for increasing the speed of quantifications of cell numbers in the CNS, called “Isotropic Fractionator” (Herculano-Houzel and Lent, 2005). This method must not be confused with stereology or counting cells on fixed tissue sections using microscopy. The Isotropic Fractionator is based on homogenizing brain tissue and counting cell nuclei in suspension using a Neubauer counting chamber or, preferably, a flow cytometer (Collins et al., 2010). However, crucial anatomical information is lost and, thus, important morphologic insights into the biology of brain diseases will be missed when using the Isotropic Fractionator (see also Young et al., 2012). For example, it is impossible to dissect the cerebral cortex of a mouse or rat into specific layers. Accordingly, quantification of the number of neurons in a specific cortical layer can be performed with stereologic cell counting (Schmitz et al., 2002) but not with the Isotropic Fractionator. Hence, this method sacrifices morphologic and neuropathologic information for a gain in execution speed, and should be used with well-defined questions in mind. Another consideration with the Isotropic Fractionator is the potential loss of cells in the dissociation and staining procedures prior to counting, requiring validation against stereological data to correct for procedural underestimation of cell yield.

In summary, stereologic cell counting remains the only method for unbiased cell quantification in histologic tissue sections. Other cell counting techniques are either biased or disregard valuable anatomical information. However, we fully expect that automated cell detection will at some point reach a stage where it can be properly integrated into stereologic cell counting. The most likely scenario will see automated methods developed for specific studies. Until then, the manual approach to stereologic cell counting is proven to be reliable and effective.

## Author contributions

Christoph Schmitz, Brian S. Eastwood, Susan J. Tappan, Jack R. Glaser, Daniel A. Peterson, and Patrick R. Hof made substantial contributions to the conception and design of the work, and to the acquisition, analysis, and interpretation of data for the work. Christoph Schmitz, Brian S. Eastwood, Susan J. Tappan, Jack R. Glaser, Daniel A. Peterson, and Patrick R. Hof drafted the work, approved the final version to be published, and agreed to be accountable for all aspects of the work in ensuring that questions related to the accuracy or integrity of any part of the work are appropriately investigated and resolved.

### Conflict of interest statement

Christoph Schmitz serves as paid consultant for MBF Bioscience (Williston, VT, USA), Jack R. Glaser is the president of MBF Bioscience, and Brian S. Eastwood and Susan J. Tappan are employees of MBF Bioscience. However, Christoph Schmitz has not received financial support directly or indirectly related to this manuscript. Patrick R. Hof and Daniel A. Peterson declare no conflict of interest.
